# Purification, characterization and thermostability improvement of xylanase from *Bacillus amyloliquefaciens* and its application in pre-bleaching of kraft pulp

**DOI:** 10.1007/s13205-017-0615-y

**Published:** 2017-04-11

**Authors:** Sharad Kumar, Izharul Haq, Jyoti Prakash, Sudheer Kumar Singh, Shivaker Mishra, Abhay Raj

**Affiliations:** 1 Environmental Microbiology Laboratory, Environmental Toxicology Group, CSIR-Indian Institute of Toxicology Research (CSIR-IITR), Vishvigyan Bhavan 31, Mahatma Gandhi Marg, Lucknow, Uttar Pradesh 226001 India; 2 Amity Institute of Biotechnology, Amity University, Lucknow Campus, Malhaur, Near Railway Station, Gomti Nagar Extension, Lucknow, Uttar Pradesh 226028 India; 3 Microbiology Division, CSIR-Central Drug Research Institute (CSIR-CDRI), Sector 10, Jankipuram Extension, Sitapur Road, Lucknow, Uttar Pradesh 226031 India; 4 Environment Management Division, Central Pulp and Paper Research Institute, Post Box 174, Paper Mill Road, Himmat Nagar, Saharanpur, Uttar Pradesh 247001 India

**Keywords:** *Bacillus amyloliquefaciens*, Xylanase, Purification, Thermostability, Kappa number, SEM

## Abstract

Xylanases have important industrial applications but are most extensively utilized in the pulp and paper industry as a pre-bleaching agent. We characterized a xylanase from *Bacillus amyloliquefaciens* strain SK-3 and studied it for kraft pulp bleaching. The purified enzyme had a molecular weight of ~50 kDa with optimal activity at pH 9.0 and 50 °C. The enzyme showed good activity retention (85%) after 2 h incubation at 50 °C and pH 9.0. This enzyme obeyed Michaelis–Menten kinetics with regard to beechwood xylan with *K*_m_ and *V*_max_ values of 5.6 mg/ml, 433 μM/min/mg proteins, respectively. The enzyme activity was stimulated by Mn^2+^, Ca^2+^ and Fe^2+^ metal ions. Further, it also showed good tolerance to phenolics (2 mM) in the presence of syringic acid (no loss), cinnamic acid (97%), benzoic acid (94%) and phenol (97%) activity retention. The thermostability of xylanase was increased by 6.5-fold in presence of sorbitol (0.75 M). Further, pulp treated with 20U/g of xylanase (20IU/g) alone and with sorbitol (0.75M) reduced kappa number by 18.3 and 23.8%, respectively after 3 h reaction. In summary, presence of xylanase shows good pulp-bleaching activity, good tolerance to phenolics, lignin and metal ions and is amenable to thermostability improvement by addition of polyols. The SEM image showed significant changes on the surface of xylanase-treated pulp fiber as a result of xylan hydrolysis.

## Introduction

Plant cell walls contain primarily three organic components, viz. cellulose, hemicellulose and lignin. Xylan is the major part of hemicellulose and a complex polysaccharide composed of a backbone of β-1, 4-glycoside-linked xylose residues. Due to the complex structure of xylan, its complete degradation requires coordinated action of several hydrolytic enzymes. Among them, xylanases (E.C. 3.2.1.8) play a crucial role in xylan hydrolysis, as it breaks 1, 4-β-d-xylosidic linkages in xylan to give short xylooligosaccharides. The xylanases are under intensive research due to their potential in food, animal feed, pulp and paper, textiles and for biofuel production (Dhiman et al. 2008). Due to emerging environmental concerns associated with chlorine use and toxicity of chlorine-bleached effluents, xylanases emerge as an attractive and environmentally safe alternative for prebleaching of kraft pulp. Its use prior to bleaching of kraft-cooked pulp has been shown reduced chlorine usage.

Most of the industrial processes are carried out at high temperature and pH in the presence of inhibitors, hence, any xylanase intended to be used for such processes must be robust enough to withstand such conditions (Bajaj and Manhas 2012) and should also be produced in a cost-effective manner so that overall cost economics is not altered. Although xylanases are produced by a wide range of different microorganisms, yet bacteria, due to their ability to grow and produce xylanases at high pH and temperature with minimum or no cellulase production, are widely exploited for xylanase production for industrial applications (Bajaj and Manhas 2012; Dhiman et al. 2008; Raj et al. 2013a). Despite the extensive search for microbial diversity for novel xylanase producers, however, xylanases with thermo and alkali stability are limited (Bajaj et al. 2011) and there remains a strong need for thermostable xylanases. Hence, both approaches requiring exploitation of microbial diversity or by mutagenesis of existing enzymes continue. Apart from this chemical modification, cross-linking, immobilization and treatment with additives have also been tried to improve the properties (Gupta 1991). The addition of polyols to protein solution provides a simple and practical approach for increasing the stability of enzymes. The polyols are thought to promote salt-bridge formation between amino acid residues, which makes the enzyme molecule more rigid and more resistant to thermal unfolding (George et al. 2001; Costa et al. 2002). However, the selection of the appropriate additive depends on the nature of the enzyme. In this study, we carried out purification and characterization of xylanase from *Bacillus amyloliquefaciens* and studied the effect of polyols on xylanase thermostability and in kraft pulp pre-bleaching.

## Materials and methods

### Chemicals and culture media

Beechwood xylan, 3, 5-dinitrosalicylic acid (DNSA), Congo red, d-xylose, alkali lignin and phenolics were purchased from Sigma (St. Louis, MO, USA). DEAE-Cellulose and sorbitol were from Merck Bioscience. All other chemicals and solvents used in this work were of analytical grade and obtained from S. D. Fine Chem. Ltd., Mumbai, India. Microbiological culture media and media ingredients were obtained from HiMedia (Mumbai, India). The wheat bran (*Triticum aestivum*) was obtained from the local market in Lucknow (U. P.), India.

### Isolation of xylanolytic bacterial isolates

Xylanolytic bacteria were isolated from the soil sample collected near Star Pulp and Paper Mill, Saharanpur, Uttar Pradesh, India, from the effluent channel. The isolation procedure was as provided: 1 g soil was added in 9 ml sterile normal saline, vortexed for one min, and 0.1 ml suspension was spread over xylan agar plate and incubated at 37 °C for 48 h. Xylan agar plates were made using basal medium containing (g/l): NaNO_3_ 3.0, K_2_HPO_4_ 0.5, MgSO_4_·7H_2_O 0.2, MnSO_4_·H_2_O 0.02, FeSO_4_·H_2_O 0.02, and CaCl_2_·2H_2_O 0.02, agar powder 15.0 and yeast extract 5.0 (Raj et al. 2013a), adjusted to pH 7.2 using 2.0% Na_2_CO_3_, and 1.0% beechwood xylan (w/v) as a source of carbon. Distinct colonies observed on xylan agar plate were re-streaked on the nutrient agar plate. The purity of isolates was checked microscopically following Gram’s staining.

### Screening of xylanase activity

Qualitative xylanase activity screening test for isolated bacterial isolates was performed by growing individual isolate on xylan agar plate at 37 °C for 48 h. The plates were stained with 1% Congo red solution for 15 min and washed with NaCl solution (1 M) to visualize xylan hydrolysis zone (Raj et al. 2013a). Quantitative assay of xylanase production was studied in the liquid basal medium by inoculating one loop full bacterial culture in 50 ml basal medium (pH 7.2) containing wheat bran (1%, w/v). The flasks were incubated at 37 °C and 120 rpm agitation. After 48 h incubation, xylanase activity was quantified in centrifuged culture supernatants. Isolate with the highest xylanase producing ability was selected for further studies.

## 16S rRNA gene sequencing

Genomic DNA was extracted and purified using GeneiPureTM Bacterial DNA purification kit (Merk India). The PCR amplification of the 16S rRNA gene was performed using 16S rRNA universal primers: 27F (5-AGAGTTTGATCCTGGCTCAG-3) and 1492R (5-TACGGTTACCTTGTTACGACTT-3) at the annealing temperature of 56 °C (35 cycles). The task of sequencing was outsourced to M/s. Amnion Bioscience (Bangalore, India). The 16S rRNA gene sequences of strain SK-3 were compared using NCBI-BLAST against the sequences of bacteria available in databanks (http://www.ncbi.nlm.nih.gov/). Program MEGA 6.0 was used to phylogenetic analysis and tree construction using Neighbour-Joining method. Some morphological and biochemical tests were also conducted (Barrow and Feltham 1993).

### Time course of bacterial growth and xylanase production

The study was conducted in liquid culture condition using the xylanase production basal medium containing wheat bran as substrate. The bacterium was cultivated in overnight (18 h) in LB broth under shaking (120 rpm) with an absorbance of 0.6 OD (A600: 1 cm cuvette) and inoculated 1% (v/v) into 500 ml of basal media containing 1.0% wheat bran. The flasks were incubated at 37 °C and 120 rpm agitation for 120 h (Innova, New Brunswick, USA). The culture broth was withdrawn at different time intervals to monitor bacterial cell growth and xylanase/cellulase activities. Cell growth was measured by taking absorbance of culture broth at 620 nm (UVvisible 2300 spectrophotometer, Techcomp, Korea).

### Xylanase assay

Extracellular xylanase/cellulase activities were assayed by measuring the released reducing sugars formed by enzymatic hydrolysis of beechwood xylan or carboxymethyl cellulose (CMC). The supernatant (8000 rpm for 10 min, at 4 °C) were assayed by procedures described by Raj et al. (2013a). The quantification of the reducing sugars released from both assays was done according to the DNS method developed by Miller (1959), using calibration curve of d-xylose and d-glucose. One unit (IU) of xylanase/cellulase activity was defined as the amount of enzyme that released 1 µM of reducing sugars equivalent to d-xylose/d-glucose per min under the assay conditions.

### Xylanase purification

The culture broth was centrifuged (8000 rpm for 20 min) after 48 h growth and filtered through 0.45 µm filters (Millex Durapore, Millipore) to remove bacterial cells. The culture supernatant (400 ml) was treated with ammonium sulfate (0–80% saturation) under constant stirring and was kept refrigerated for 2 h. Afterwards, it was centrifuged and the pellet was dissolved in 0.05 M sodium phosphate buffer (pH 8.0) and dialyzed at 4 °C for overnight against the same buffer using 12 kDa cut-off membrane (Himedia, LA395-5MT). The dialyzed enzyme solutions were pooled, desalted, and concentrated by ultrafiltration using Amicon Ultra-15 10 kDa (Millipore). Concentrated enzyme (5 ml) was applied to an ion-exchange column (1.5 cm × 30 cm) packed with DEAE-cellulose ion exchange column equilibrated with the same buffer. Proteins were eluted first with 20 ml 0.05 M sodium phosphate buffer to remove the unbound proteins and then with a 0.1–1.0 M NaCl gradient at a flow rate of 30 ml/h. All the steps were carried out at 4–8 °C. The chromatographic elutes were assayed for protein and xylanase activity. The protein concentration was determined either by measuring the absorbance at 280 nm or Lowry’s method using BSA as a standard (Lowry et al. Lowry et al. 1951).

### SDS-PAGE and zymography

The ammonium sulfate precipitated and DEAE-cellulose purified enzymes were concentrated using ultrafiltration (Amicon Ultra-15 10 kDa, Millipore) and used for SDS-PAGE zymography (Tseng et al. 2002). Samples (25–30 µg protein) were subjected to SDS-PAGE using 10% polyacrylamide in the gel. Electrophoresis was carried out using Mini-Gel Electrophoresis unit (Microkin, Techno Source, Mumbai, India). The samples were loaded in duplicate without the addition of β-mercaptoethanol (Raj et al. 2013a). On completion of electrophoresis, the gel was cut in two parts. One part of the gel was used for Coomassie brilliant blue R-250 staining and the other portion was used for zymography. Zymogram analysis was performed using the basic protocol of Tseng et al. (2002). The gel was washed twice for 30 min at 4 °C in 50 mM sodium phosphate buffer (pH 7.0) containing isopropanol (25%). Afterwards, it was incubated in the same buffer solution containing 1.0% beechwood xylan solution at 37 °C for 30 min, staining and de-staining of the gel was performed using 0.1% Congo red and 1 M NaCl, respectively. Decolourization of gel around the protein bands was correlated with enzyme activity. The molecular weight of proteins was determined by comparing them with standard protein marker (BlackBio Biotech India).

### Effect of pH and temperature on activity

Effect of pH on xylanase activity was estimated by incubating the purified enzyme at 50 °C for 15 min in 1.0% (w/v) beechwood xylan solution prepared in 100 mM buffer. The buffer solutions used for the study were citrate buffer (pH 4.0–6.0), phosphate buffer (pH 6.0–8.0), tris–HCl buffer (pH 8.0–9.0), and glycine–NaOH buffer (pH 9.0–11.0). The effect of temperature on xylanase activity was determined by incubating the enzyme with 1.0% (w/v) beechwood xylan prepared in tris–HCl buffer (pH 9.0) for 15 min at 30, 40, 50, 60, 70, 80, 90, and 100 °C. Thermostability of xylanase was determined by pre-incubating the enzyme between 30 to 80 °C temperature in tris–HCl buffer (pH 9.0) for 3 h, afterwards, residual activity was measured at 50 °C.

### Kinetic determinations

The kinetic studies of the enzymes were determined by measuring the initial hydrolysis rate of beechwood xylan at different substrate concentrations (1–20 mg/ml) prepared in 100 mM tris–HCl buffer, pH 9.0 at 50 °C for 10 min. The Michaelis–Menten constant (*K*_m_), the maximum velocity (*V*_max_) and *K*_cat_ were calculated using Lineweaver–Burk plots with the help of Graphpad Prism software7.0 (Lineweaver and Burk 1934).

### Effect of metal ions and additives on enzyme activity

The effects of different metal ions (Mn^2+^, Ca^2+^, Fe^2+^, Zn^2+^, Mg^2+^, Cu^2+^ and Hg^2+^) and additives (β-mercaptoethenol, DTT, EDTA and SDS) on xylanase activity were investigated by including them in the reaction mixtures at the final concentration of 2, 4, 6, 8 and 10 mM, respectively. The effect of lignin (0.25–1.0 mg/ml) and phenolics (syringic acid, benzoic acid and cinnamic acid and phenol) were also investigated at the final concentration of 2 mM. The enzyme activity assays were performed at 50 °C in tris–HCl buffer (pH 9.0). The enzyme activity without metal ions/additives was treated as control and considered as 100%.

### Effect of polyols on xylanase thermostability

Effect of polyols such as sorbitol, mannitol and glycerol on thermostability was studied by adding them in the enzyme assay mixture at the final concentration of 0.5 M. The incubation was performed at 70 °C for 3 h and aliquots were withdrawn after every 30 min. The residual xylanase activity was assayed under optimum condition (50 °C and pH 9.0). The stability of the enzyme was expressed as a percentage of residual activity compared to the initial enzyme activity. The polyol which showed thermostability enhancement was further studied over a concentration ranged from 0.25 to 1.0 M at 70 °C.

### Pulp pre-bleaching studies

Pulp pre-bleaching studies were conducted on hardwood unbleached kraft pulp collected from Star Paper Mill, Saharanpur, (Uttar Pradesh, India). Oven dried unbleached kraft pulp was washed extensively to remove the alkali. The pulp prebleaching studies were performed at pH 9.0 and 60 °C with a xylanase dose of 20 IU/g added to oven dried pulp of 10% consistency and incubated for 3 h in a water bath under shaking at 100 rpm. Pulp prebleaching studies were also conducted in presence of 0.75 M sorbitol. Pulp without enzyme treatment was taken as control. Control and enzyme treated pulp samples were filtered, washed with tap water, and dried in an oven at 70 °C to a constant weight. Reducing sugars released from untreated and enzyme-treated pulp were measured according to Miller (1959). The release of the phenolics and hydrophobic compounds were measured at 237 and 465 nm, respectively (Gupta et al. 2000; Patel et al. 1993). Kappa number, (lignin content in pulp), was estimated by reaction of pulp with acidified potassium permanganate (TAPPI 1985).

### Scanning electron microscopy (SEM) analysis

Surface morphology of enzyme treated and untreated pulp samples were examined by scanning electron microscope (SEM, QUANTA 450 FEG, FEI, Netherland). Untreated and treated pulp samples are oven dried by incubating at 70 °C overnight. The samples were placed on a conducting carbon tape over aluminium stubs and coated with platinum in a sputter coater (SC 7620, Quorum Technology Ltd, UK). SEM images of treated and untreated pulp were taken at 1000× magnifications at an accelerating voltage of 10 kV.

## Results and discussion

### Isolation and characterization of xylanase producer

**Fig. 1:**
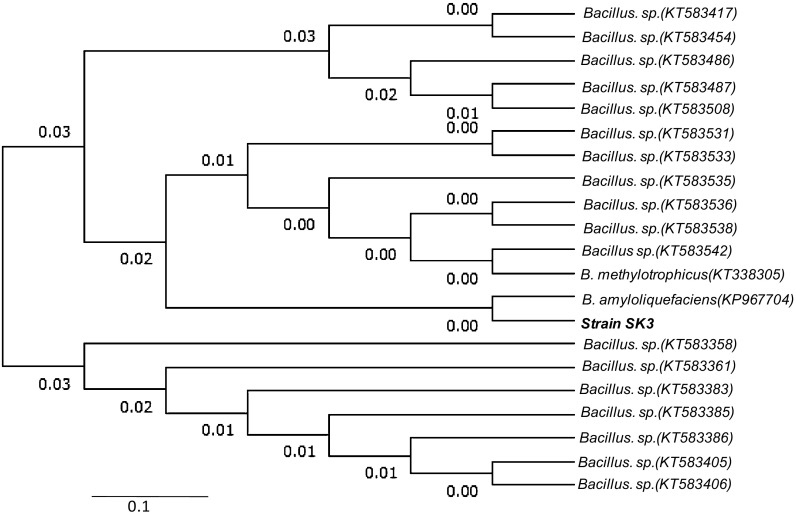
Phylogenetic tree based on a comparison of 16S rDNA sequences of xylanase-producing strain SK-3. The phylogenetic tree was constructed on the aligned datasets using neighbour joining (NJ) method using the program MEGA 6.0. Their names and respective accession numbers are given in the tree

Total five xylanolytic bacterial isolates (named as SK 1-5) were isolated from paper mill effluent contaminated soil. All bacterial isolates formed clear zones on xylan agar plate ranging from 1.3 to 2.5 cm (data not shown) and produced xylanase utilizing wheat bran as substrates. The mean xylanase production by different isolates ranged from 6.7 to 42.5 IU/ml after 48 h. Highest xylanase production was recorded for strain SK-3 (42.5 ± 2.5) followed by SK-2 (12.4 ± 1.6), SK-4 (8.8 ± 1.4) and SK-5 (6.7 ± 1.5) and SK-1 (4.5 ± 1.2). Being a highest xylanase producer, strain SK-3 was selected and characterized by 16S rRNA gene sequence analysis. The nucleotide (1341 bp) blast analysis of 16S rDNA sequence showed 99% identity with several *Bacillus sp*., however, maximum homology (100%) was observed with *Bacillus amyloliquefaciens* (Fig. 1). Accordingly, strain SK-3 was named as *B. amyloliquefaciens*. 16S rDNA sequence has been deposited in NCBI GenBank (Accession no. KU877335). Strain SK-3 was aerobic, rod-shaped gram-positive bacterium and showed a positive reaction for catalase, oxidase, amylase and motility. It was indole negative.

### Time course profile of growth and xylanase production by *B. amyloliquefaciens*

**Fig. 2:**
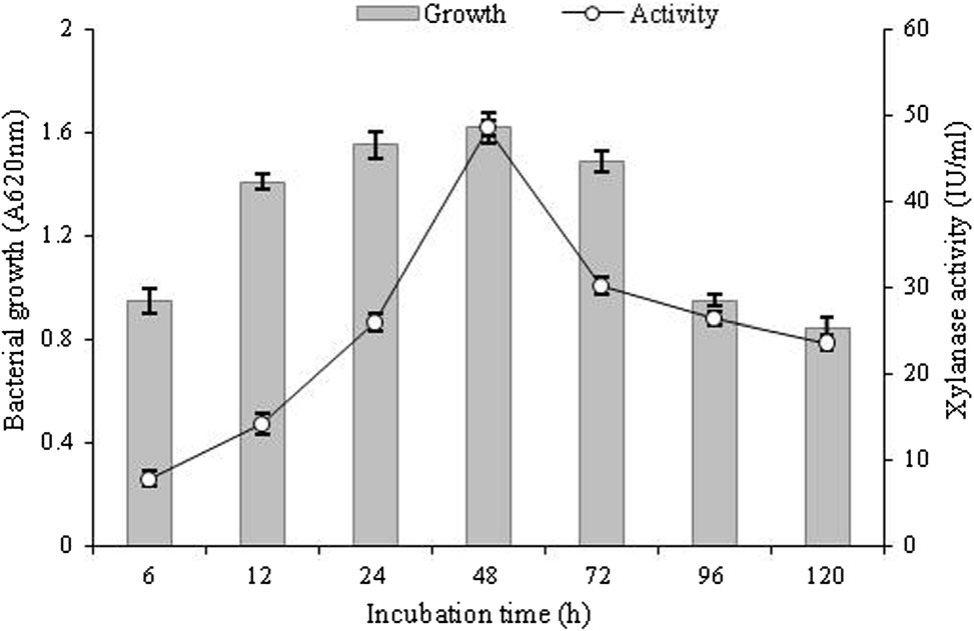
Time course of growth and xylanase production by *B. amyloliquefaciens* strain SK-3 in basal medium containing wheat bran (1% w/v) at 37 °C, 120 rpm and pH 7.2. Experiments were performed in triplicate and results are mean ± SD of three values

Time-dependent growth and xylanase production study were performed in liquid basal medium (pH 7.2) containing 1% wheat bran at 37 °C and 120 rpm for 120 h. The results showed (Fig. 2) increasing growth and xylanase production by *B. amyloliquefaciens* strain SK-3 with time. Maximum bacterial growth (OD_620nm_ 1.6) and xylanase activity (48.5 ± 1.8 IU/ml) were observed at 48 h. Further, no cellulase activity was determined using CMC as a substrate. This revealed that *B. amyloliquefaciens* strain SK-3 xylanase was cellulase-free xylanase. Amore et al. (2015) isolated *B. amyloliquefaciens* from the soil sample of Western Ghat region and this isolate produced maximum 10.5 U/ml xylanase on brewer’s spent grain substrate after 24 h. Breccia et al. (1998) isolated *B. amyloliquefaciens* from soil sample showed xylanase activity of 10.4 U/ml on birchwood xylan after 48 h. The production of xylanase by current isolate is significantly higher than those reported earlier. Wheat bran had been used earlier also for both submerged fermentation (SmF) and solid state fermentation (SSF) based xylanase production (Raj et al. 2013b; Sanghi et al. 2010). Wheat bran being an agro-industrial residue it helps to bring down the production cost.

### Purification of *B. amyloliquefaciens* xylanase, SDS-PAGE and zymography

The *B. amyloliquefaciens* strain SK-3 xylanase purification was performed initially by ammonium sulphate precipitation followed by DEAE-Cellulose chromatography. The xylanase obtained after ammonium sulphate precipitation contained 3.5 mg/ml protein and showed 295 IU/ml activity was loaded on DEAE-cellulose column. The eluted fraction numbers 24–26 showing maximum activity were pooled together for further studies. The final purification was 5.06 fold with 22.12% recovery (Table 1). The specific activity was 217.39 IU/mg protein. There is wide variation in the specific activity of xylanase from different bacterial species. The specific activity of purified xylanase from *B. amyloliquefaciens* strain SK-3 in the present study was higher than the previous reports (Sharma et al. 2013; Bajaj and Manhas 2012; Mishra and Thakur 2010). Kamble and Jadhav (2012) reported xylanase from *Bacillus arseniciselenatis* strain DSM-15340 having high specific activity of 299.25 IU/mg protein. However, this is active at neutral pH 8.0.

**Table 1:** Summary of the purification steps of an extracellular xylanase produced by *B. amyloliquefaciens* strain SK-3

Purification step	Volume (ml)	Total activity (IU)	Total protein (mg)	Specific activity (IU/mg)	Recovery (%)	Purification fold
Culture filtrate	400	11,300	263.50	42.88	100	1.0
Crude AMS	30	8850	105.45	84.28	78.31	1.96
DEAE-cellulose	9	2500	11.50	217.39	22.12	5.06

**Fig. 3:**
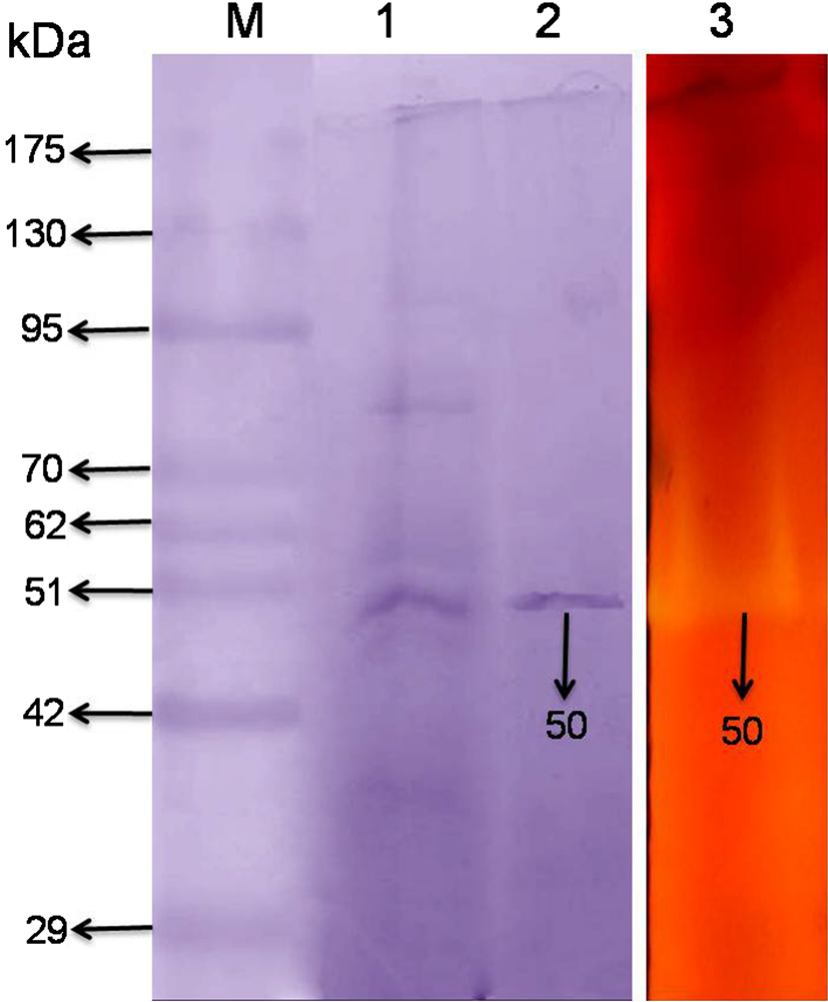
SDS-PAGE and zymogram analysis of crude and purified *B. amyloliquefaciens* strain SK-3. *Lane M* protein marker, *Lane 1* AMS precipitated crude xylanase and *Lane 2* DEAE cellulose purified xylanase. *Lane 3* zymogram of DEAE-cellulose purified xylanase

Xylanase separated by SDS-PAGE gel was treated for enzyme renatuaration (Tseng et al. 2002) followed by zymogram analysis which showed a single protein band corresponding to ~50 kDa with clear decolourisation zone (Fig. 3), suggesting positive xylanase activity of purified protein. The molecular weight (MW) of purified xylanase was comparable to earlier reported xylanase from *Streptomyces* sp. (40 kDa) (Mander et al., 2014). Usually, MW of xylanases reported from different bacteria varies between 20 and 145 kDa (Raj et al. 2013b; Kulkarni et al. 1999) however; a higher MW xylanase (340 kDa) had also been reported (Sá-Pereira et al. 2002).

### Biochemical properties of purified xylanase

**Fig. 4:**
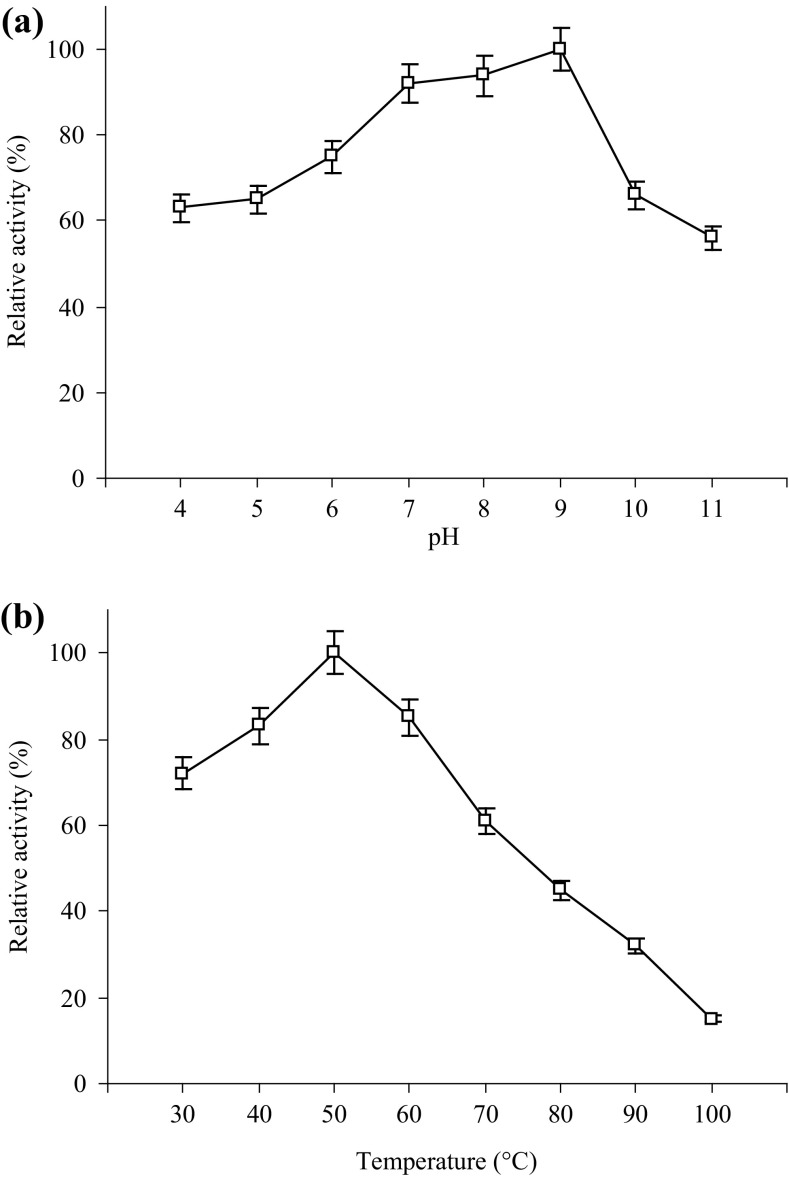
Optimum pH (**a**) and temperature (**b**) for the activity of purified xylanase from *B. amyloliquefaciens* strain SK-3. Experiments were performed in triplicate and results are mean ± SD of three values

The activity of the purified xylanase at different pH values and at 50 °C temp is shown in Fig. 4a. The optimum xylanase activity was at pH 9.0, while it showed good activity retention at 66 and 56% at pH 10 and 11, respectively. These results are consistent with those reported for xylanases from *S. maltophilia and B. halodurans* (Raj et al. 2013b; Kumar and Satyanarayana 2011). Although xylanases with optimum activity at pH 9.0 had been reported earlier; however, significant activity losses were observed at higher pH (Bajaj and Manhas 2012; Nakamura et al. 1993). The xylanase being reported in this study is superior in its activity retention compared to earlier reported xylanases.

The activity at different temperatures at pH 9.0 is shown in Fig. 4b. The enzyme shows activity at different temperatures with optimum being at 50 °C. Almost 85 and 61% activity was observed at 60 and 70 °C, respectively. Similar temperature optimum of 50 °C was observed for xylanase from *Bacillus* sp. (Nakamura et al. 1993).

**Fig. 5:**
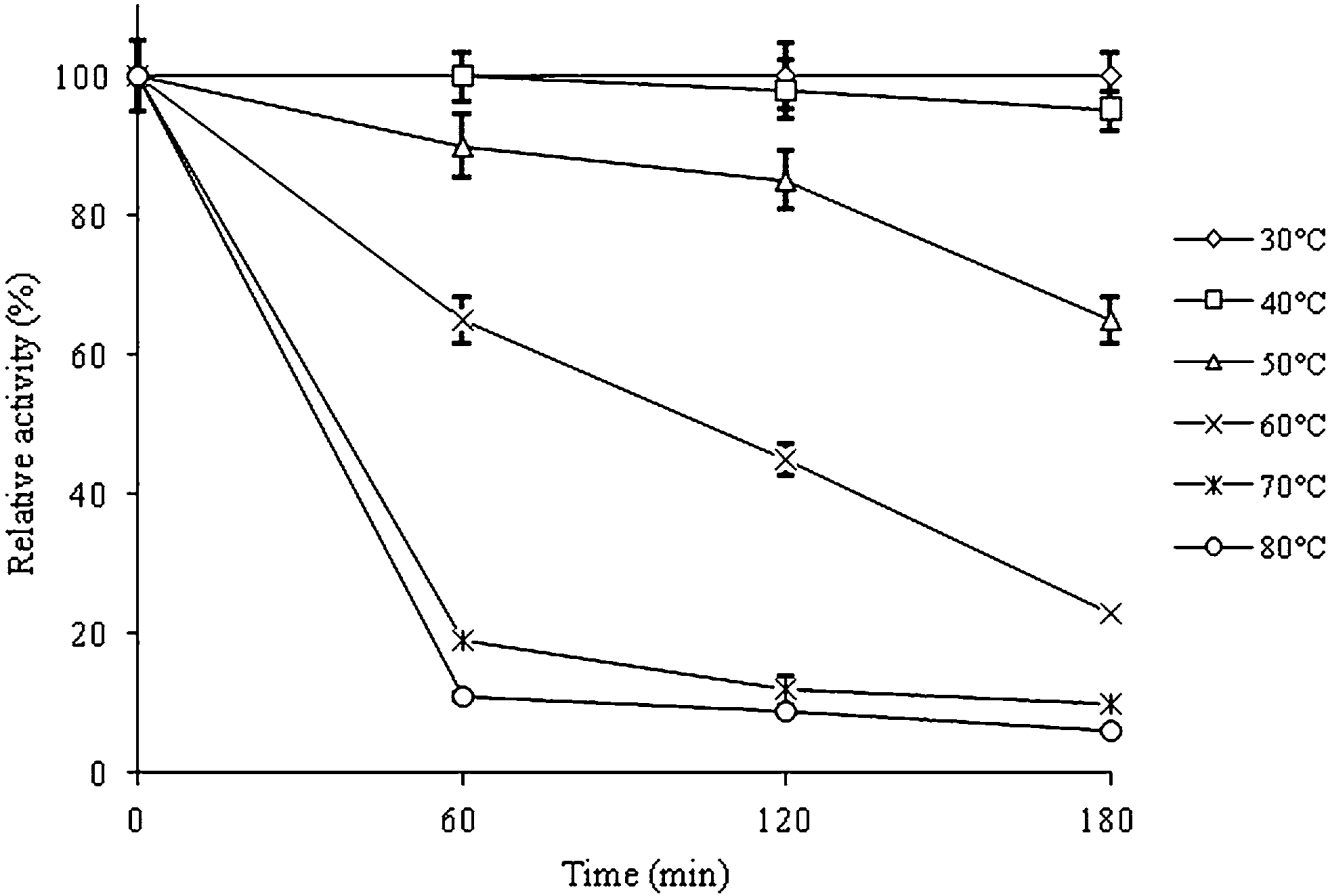
Thermostability of the purified xylanase from *B. amyloliquefaciens* strain SK-3 at different temperatures at pH 9.0. Experiments were performed in triplicate and results are mean ± SD of three values

Thermostability of xylanase was evaluated by pre-incubating the enzyme at the temperature ranging from 30 to 80 °C at pH 9.0 for 180 min (Fig. 5). The enzyme showed approximately 65% activity retention was observed at 50 °C after 180 min of exposure. Also, at 70 and 80 °C, it retained 10 and 6% activity (Fig. 5). The loss of enzyme activity throughout the elevated temperature ranges is related to changes of the enzyme conformation (Cui et al. 2008). These results are consistent with thermal stabilities of other bacterial xylanases reported in the literature. Xylanase from *Bacillus* sp. strain 41-M1 showed 90% loss of the activity after 30 min incubation at 60 °C and pH 9.0 (Nakamura et al. 1993), while xylanase from *Bacillus* sp. VI-4 retained approximately 15–20% activity at 60 °C and pH 9.0 after 30 min incubation (Yang et al. 1995). In comparison, xylanase from present study shows a better thermostability profile with 65, 45 and 23% activity retention at 60 °C after 60, 120, and 180 min incubation.

The *K*_m_, *V*_max_ and *K*_cat_ values were determined by the Lineweaver–Burk double reciprocal plot using different concentration of beechwood xylan as substrate and maximum activity was observed in 10 mg/ml concentration (data not shown). The determined *K*_m_, *V*_max_ and *K*_cat_ values of the enzyme were 5.6 mg/ml, 433 μM/min/mg proteins and 106.1 (min^−1^) respectively. The *K*_m_ value for the xylanase is low which shows that the xylanase has better affinity with substrate beechwood xylan substrate. The *K*_m_ and *V*_max_ value of the enzyme were 4.4 mg/ml and 287 U/mg from *Bacillus* sp. (Mishra and Thakur 2010). Sanghi et al. (2010) also reported that the *K*_m_ and *V*_max_ in birch wood xylan were 3.33 mg/ml and 100 IU/ml respectively from *Bacillus subtilis* ASH.

The effects of various metal ions and additives at the different concentration on xylanase activity are summarized in Table 2. Metal ions Mn^2+^, Ca^2+^ and Fe^2+^ showed different levels of stimulatory effects on the activity of the xylanase, and the stimulatory effects were gradually enhanced with increasing concentrations. The relative activity of xylanase was shown to be enhanced up to 40 and 45% by Mn^2+^, Ca^2+^, respectively at 10 mM. No significant effects on enzyme activity were observed in presence of Zn^2+^ and Mg^2+^ ions at each concentration. However, xylanase activity was strongly inhibited by Cu^2+^ and Hg^2+^ in a dose-dependent manner and causing 78 and 88% inhibition at 10 mM. The inhibition or stimulation of enzyme activity may be due to metal ions interaction with SH or carboxyl groups which led to an altered conformation of protein subsequent inactivation (Nagar et al. 2012). Lee et al. (2008); Park and Cho (2010) reported the stimulatory effect of Mn^2+^ and Ca^2+^ on xylanase of *B. licheniformis* and *Paenibacillus* sp. strain K1J1. Hg^2+^ and Cu^2+^ have previously been reported to strongly inhibit the activity of xylanase from *B. licheniformis* and *B. pumilus* (Bajaj and Manhas 2012; Nagar et al. 2012). Inhibition by Hg^2+^ ions may be due to its interaction with sulphydril groups, suggesting that there is an important cysteine residue in or close to the active site of the enzyme (Bastawde 1992). Metal ion Cu^2+^ has a strong affinity for amino acids and carboxyl groups and may affect the enzyme activity by its interactions with these groups (Sanghi et al. 2010; Menon et al. 2010). β-Mercaptoethanol and DTT also stimulated xylanase activity and stimulation was dose-dependent. This stimulation of enzyme activity in the presence of DTT (34%) and β-mercaptoethanol (14%) could be due to the protection of oxidation of sulfhydryl groups (Knob and Carmona 2009). EDTA and SDS showed strong inhibitory effects on xylanase activity in a dose-dependent manner and causing 58 and 68% inhibition at 10 mM. The activity inhibition by a metal chelator (EDTA), indicating that the enzyme requires metal ions for its action (Knob and Carmona 2009). Inhibition of xylanase activity by SDS suggests presence of hydrophobic interactions in maintaining xylanase structure. Similar to our results, the xylanase activity of *Paenibacillus campinasensis* G1-1 was inhibited in presence of EDTA and SDS with increasing concentrations (Hongchen et al. 2012).

**Table 2:** Effect of different metal ions and additives on activity of purified xylanase from *B. amyloliquefaciens* strain SK-3

Additive metal ions	Relative xylanase activity (%)				
	2 mM	4 mM	6 mM	8 mM	10 mM
None	100 ± 1.2	100 ± 1.6	100 ± 2.7	100 ± 1.6	100 ± 1.5
Mn^2+^	120 ± 2.5	126 ± 2.0	130 ± 2.2	135 ± 2.6	140 ± 1.8
Ca^2+^	116 ± 1.5	120 ± 1.8	124 ± 2.4	131 ± 2.6	145 ± 1.4
Fe^2+^	102 ± 2.0	102 ± 1.0	105 ± 1.6	108 ± 2.4	110 ± 2.0
Zn^2+^	95 ± 1.6	94 ± 0.8	92 ± 1.2	90 ± 1.6	88 ± 1.4
Mg^2+^	94 ± 1.8	92 ± 1.4	92 ± 1.8	90 ± 1.1	90 ± 0.9
Cu^2+^	68 ± 1.4	60 ± 1.2	54 ± 2.1	37 ± 0.8	22 ± 1.0
Hg^2+^	60 ± 0.6	52 ± 0.8	44 ± 1.0	30 ± 0.8	12 ± 1.2
Inhibitors					
None	100 ± 1.2	100 ± 1.6	100 ± 2.2	100 ± 1.8	100 ± 1.2
β-Mercaptoethanol	105 ± 2.8	109 ± 2.2	118 ± 1.8	125 ± 2.0	134 ± 1.4
DTT	102 ± 1.2	106 ± 1.8	108 ± 2.0	110 ± 1.6	114 ± 1.4
EDTA	75 ± 0.6	71 ± 0.8	65 ± 0.4	55 ± 0.6	42 ± 0.9
SDS	80 ± 1.2	75 ± 1.6	64 ± 1.8	52 ± 1.0	32 ± 1.4

Kraft pulp contains residual lignin which may affect xylanase activity hence; effect of lignin on xylanase activity was studied in a concentration range of 0.25–1.0 mg/ml (Table 3). No effect of lignin on activity was observed at lower lignin concentration (0.5 mg/ml). However, higher concentrations caused partial inhibition of the enzyme activity and almost 92 and 90% of its original activity was retained at 0.75 and 1.0 mg/ml, respectively. An earlier study by Morrison et al. (2011) to study the effect of soluble lignin on xylanase activity observed 25% inhibition at low lignin concentration of 0.075 mg/ml. However, Kaya et al. (2000) observed an increased xylanase mediated hydrolysis (20%) with increasing lignin concentration (0–0.06%). The kraft pulp contains trace amounts of low molecular weight phenolics which may affect the enzyme activity. The phenolic compounds can be coming from either lignin degradation or are naturally present in plants. Xylanase activity in presence of different phenolic compounds (2 mM) is presented in Table 3. Slight increase in xylanase activity in the presence of syringic acid and a decrease with benzoic acid, cinnamic acid and phenol was observed. Inhibition of xylanase activity was reported by Morrison et al. (2011) in presence of coumaric acid and gallic acid.

**Table 3:** Effect of lignin and phenolics on activity of purified xylanase from *B. amyloliquefaciens* strain SK-3

Compounds	Relative xylanase activity (%)
Lignin (mg/ml)	
None	100 ± 0.8
0.25	100 ± 0.8
0.50	100 ± 2.0
0.75	92 ± 1.0
1.0	90 ± 0.8
Phenolics (2 mM)	
None	100 ± 0.4
Syringic acid	106 ± 0.5
Benzoic acid	94 ± 1.5
Cinnamic acid	97 ± 1.8
Phenol	97 ± 1.3

### Effect of polyols on xylanase thermostability

**Fig. 6:**
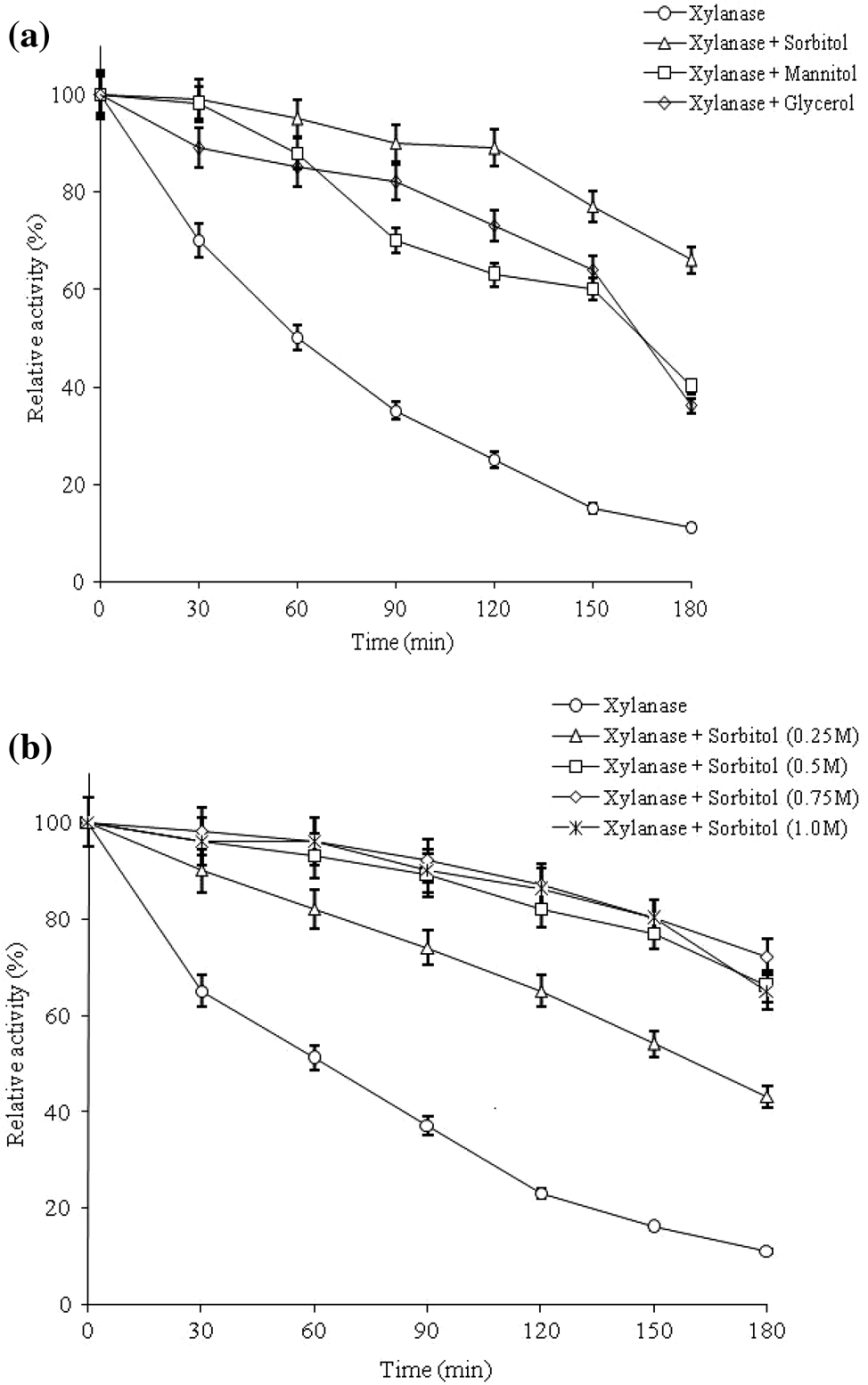
Effect of polyols on thermostability of the purified xylanase from *B. amyloliquefaciens* strain SK-3 cultivated in basal medium containing 1% wheat bran. **a** The enzyme solutions were pre-incubated in presence of sorbitol, mannitol and glycerol at concentration of 0.5 M prior to enzyme assay. **b** The enzyme solutions were incubated under same condition as in (**a**) with presence of sorbitol of 0.25, 0.5, 0.75 and 1.0 M prior to enzyme assay at optimal conditions. Experiments were performed in triplicate and results are mean ± SD of three values

It was found that purified xylanase was quite stable (retaining 65%) at least for 180 min at 50 °C and pH 9.0. In order to avoid this thermal inactivation, thermostability of xylanase in the presence of 0.5 M sorbitol, mannitol and glycerol were investigated at 70 °C. Figure 6a shows that all sugars have increased the stability of xylanase by several folds over control. The highest stability increase was observed in the presence of sorbitol (6.0-fold) followed by mannitol (3.6-fold) and glycerol (3.3-fold) after 180 min (3 h) and compared activity in control (11%), enzyme provided with polyols retained almost 66, 40 and 36% of its original activity in the presence of sorbitol, mannitol and glycerol, respectively. Also, a concentration dependent effect of sorbitol on activity was observed (Fig. 6b) and in the presence of 0.25, 0.5, 0.75 and 1 M sorbitol, an increase of 3.9, 6.0, 6.5, and 5.9 fold xylanase activities was observed over control after 3 h incubation at 70 °C. Earlier studies had also reported a positive effect of sorbitol on xylanases stability (George et al. 2001; Khandeparkar and Bhosle 2006; Bankeeree et al. 2014). The Phenomenon of protein stabilization by polyols may be due to changes in enzyme microenvironment resulting in a more rigid conformation of the enzyme (Lemos et al. 2000). The stabilizing effect of additives is not an absolute effect valid for all enzymes, and depends on the nature of the enzyme, on its hydrophilic and hydrophobic character and on the degree of interaction with the additive (George et al. 2001). However, the improvement of xylanase stability in the presence of sorbitol suggests its applicability in the pulp-bleaching process.

### Pre-bleaching of kraft pulp

**Fig. 7:**
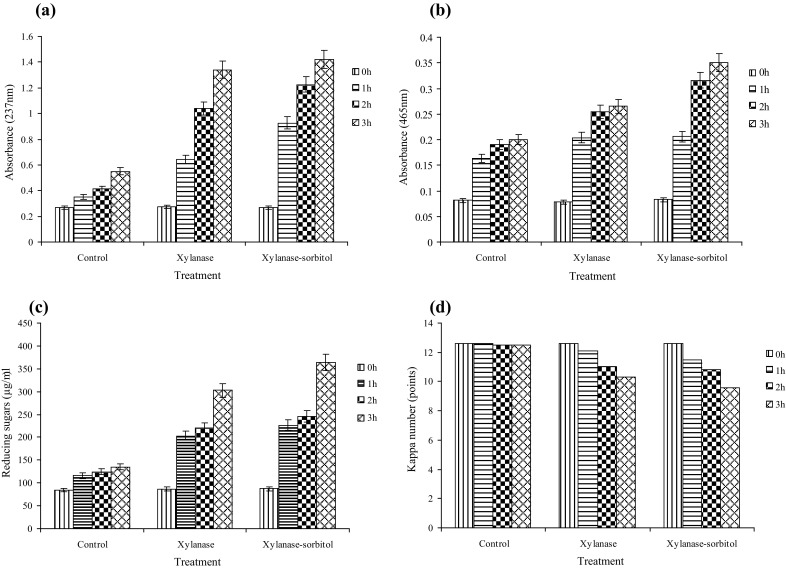
Effect of the xylanase pre-bleaching on the release of hydrophobic compounds (**a**), phenolic compounds (**b**), reducing sugars (**c**) and kappa number reduction (**d**) of kraft pulp. Experiments were performed in triplicate and results are mean ± SD of three values

The effect of xylanase on kraft Pulp pre-bleaching studies was performed using purified xylanase added with and without sorbitol (0.75 M) at 60 °C and pH 9.0. Details about the release of reducing sugars, phenolics and other hydrophobic compounds and reduction of kappa number at different time points are presented in Fig. 7. The filtrate of pulp treated with xylanase and xylanase-sorbitol mix had 2.4, 2.6 and 1.3, 1.8 fold higher phenolic and hydrophobic compounds, respectively than the filtrate of xylanase untreated pulp (Fig. 7a, b). The amount of reducing sugar was treatment time dependent. The initial amount of reducing sugars in the filtrate of untreated pulp was 135 µg/ml. The reducing sugar content after treatment with xylanase and xylanase-sorbitol increased to 302.1 and 364.2 µg/ml, respectively (Fig. 7c). This increase in reducing sugars content correlates positively with xylanase mediated xylan degradation. Kappa number, which measures the amount of lignin present in the pulp was 12.6 for untreated pulp. After treating it with 20 U/g of xylanase and xylanase-sorbitol, it decreased to 10.3 and 9.6, respectively after 3 h. This suggests a decrease of 18.3 and 23.8% of kappa number after treatment with xylanase and xylanase-sorbitol, respectively (Fig. 7d). The release of phenolics and hydrophobic compounds and the reduction in kappa number coupled to the release of reducing sugars suggest the dissociation of lignin-carbohydrate complex (LCC) from the pulp fibers by enzyme action (Khandeparkar and Bhosle 2007). Earlier studies with xylanases show a decrease in kappa number after treatment. The *Antherobacter* sp. MTCC 5214 xylanase showed 20% reduction in kappa number of kraft pulp after 2 h treatment (Khandeparkar and Bhosle 2007), while *B. pumilus* xylanase showed 14% reduction in kappa number of kraft pulp (Bim and Franco 2000). In the present study, we observed a better reduction (23.8%) in kappa number after treatment with purified xylanase-sorbitol mix.

### SEM of xylanase treated pulp

**Fig. 8:**
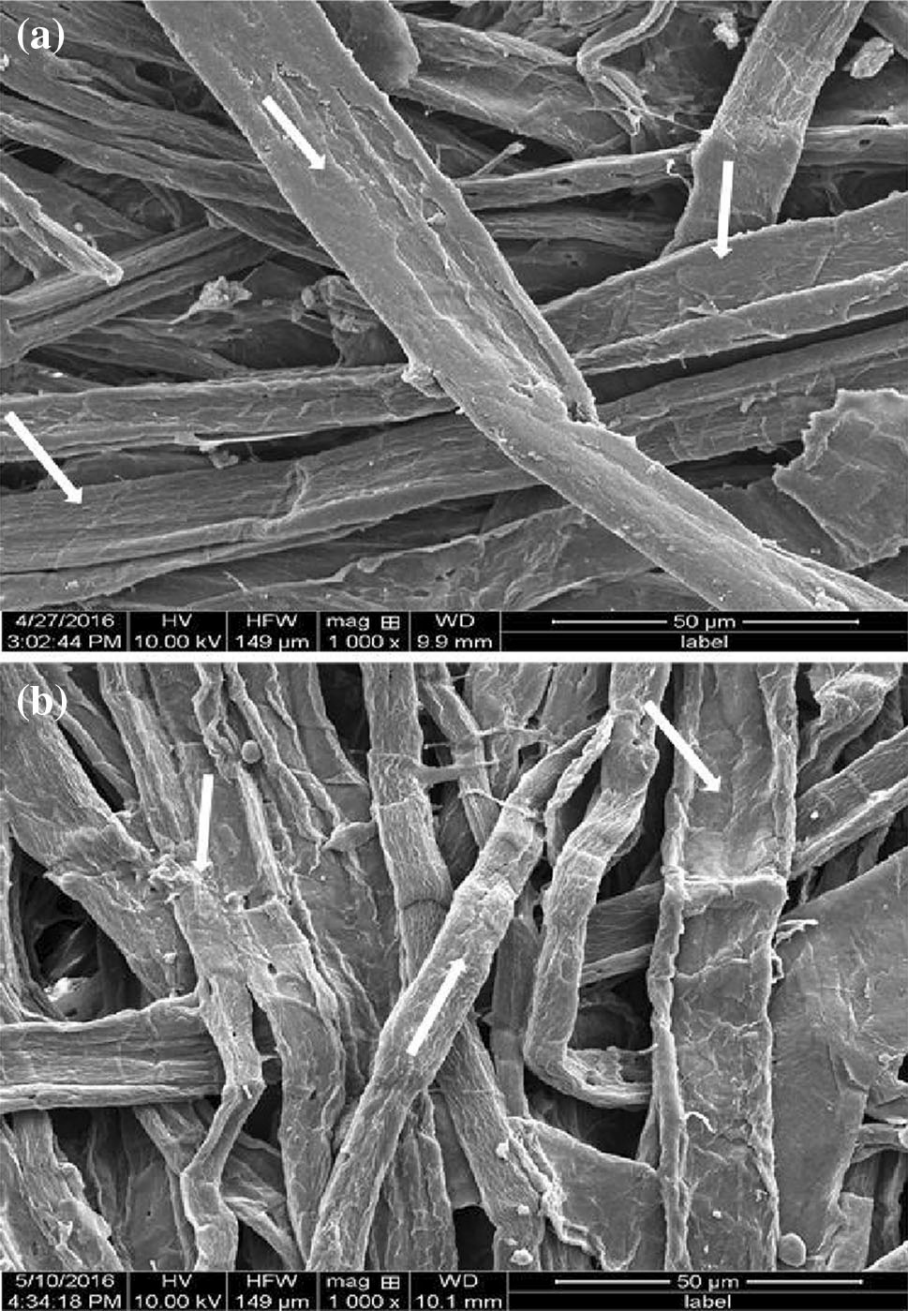
SEM images of the untreated (**a**) and xylanase treated (**b**) pulp fiber at ×1000 magnification

Scanning electron microscopy analysis of pulp fibers was carried out to observe the morphological changes after xylanase treatment. SEM images of untreated and xylanase treated pulp (Fig. 8a, b) showed change in morphology of pulp fibers after treatment with xylanase. The surface of untreated pulp fibers surface was smooth (Fig. 8a), whereas that of the treated fibers was rough (Fig. 8b). Further, the xylanase-treated image showed an increase in swelling, peeling and loosening of pulp fibers. These changes on the surface of xylanase treated pulps suggest hydrolysis of xylan in pulp (Fig. 8b). Similar observations on pulp fiber after enzyme treatment have been reported (Nagar et al. 2013).

## Conclusions

The findings of the present study suggest that xylanase from *B. amyloliquefaciens* strain SK-3 was cellulase-free with estimated MW of 50 kDa. The optimum pH and temperature for the purified xylanase were pH 9.0 and 50 °C. The enzyme shows good activity retention under alkaline pH. Enzyme activity was stimulated by Mn^2+^ and Ca^2+^ metal ions. The thermostability of the xylanase improved by 6.5-fold at 70 °C, after sorbitol addition. The xylanase produced by present strain showed better reduction of kappa number (23.8%) compared to earlier studies. The sorbitol serves as a potential stabilizer for xylanase from *B. amyloliquefaciens* strain SK-3, which may be of commercial use in industries including pulp and paper industry.
